# Within-Compound Versus Public Latrine Access and Child Feces Disposal Practices in Low-Income Neighborhoods of Accra, Ghana

**DOI:** 10.4269/ajtmh.17-0654

**Published:** 2018-03-19

**Authors:** Rebecca Lyn Ritter, Dorothy Peprah, Clair Null, Christine L. Moe, George Armah, Joseph Ampofo, Nii Wellington, Habib Yakubu, Katharine Robb, Amy E. Kirby, Yuke Wang, Katherine Roguski, Heather Reese, Chantal A. Agbemabiese, Lady Asantewa B. Adomako, Matthew C. Freeman, Kelly K. Baker

**Affiliations:** 1 College of Public Health, University of Iowa, Iowa City, Iowa;; 2 Center for Global Safe Water, Sanitation, and Hygiene, Emory University, Atlanta, Georgia;; 3 London School of Hygiene and Tropical Medicine, London, United Kingdom;; 4 Mathematica Policy Research, Washington, District of Columbia;; 5 Noguchi Memorial Institute for Medical Research, University of Ghana, Accra, Ghana;; 6 Council for Scientific and Industrial Research Water Research Institute, Accra, Ghana;; 7 Training Research and Networking for Development (TREND), Accra, Ghana;; 8 Department of Environmental Health, Emory University, Atlanta, Georgia

## Abstract

In crowded urban settlements in low-income countries, many households rely on shared sanitation facilities. Shared facilities are not currently considered “improved sanitation” because of concerns about whether hygiene conditions sufficiently protect users from the feces of others. Prevention of fecal exposure at a latrine is only one aspect of sanitary safety. Ensuring consistent use of latrines for feces disposal, especially child feces, is required to reduce fecal contamination in households and communities. Household crowding and shared latrine access are correlated in these settings, rendering latrine use by neighbors sharing communal living areas as critically important for protecting one’s own household. This study in Accra, Ghana, found that household access to a within-compound basic latrine was associated with higher latrine use by children of ages 5–12 years and for disposal of feces of children < 5 years, compared with households using public latrines. However, within-compound access was not associated with improved child feces disposal by other caregivers in the compound. Feces was rarely observed in household compounds but was observed more often in compounds with latrines versus compounds relying on public latrines. *Escherichia coli* and human adenovirus were detected frequently on household surfaces, but concentrations did not differ when compared by latrine access or usage practices. The differences in latrine use for households sharing within-compound versus public latrines in Accra suggest that disaggregated shared sanitation categories may be useful in monitoring global progress in sanitation coverage. However, compound access did not completely ensure that households were protected from feces and microbial contamination.

## INTRODUCTION

An estimated 1.7 billion episodes of diarrhea occur in children less than 5 years of age globally each year, 437 million of which occur in sub-Saharan Africa alone.^1^ Furthermore, 10% of all deaths worldwide in this age group are attributed to this diarrheal disease burden.^2^ The greatest risk factors for diarrheal diseases in low-income countries are poor sanitation, water, and hygiene conditions.^3,4^ Interventions that improve household sanitation access are considered cost-effective strategies for reducing fecal contamination in the environment and preventing the spread of gastrointestinal disease.^5,6^ The Sustainable Development Goals (SDGs) have targeted the elimination of open defecation by 2025, with all people using adequate household sanitation facilities by 2040.^7^ Progress toward these goals is measured by the World Health Organization and UNICEF Joint Monitoring Program (JMP) through the percentage of the population living in households where “improved” sanitation facilities protect users from exposure to the feces of other individuals by installing a barrier between users and human excreta.^8^ “Safely managed” and “basic” household access to a private improved facility is considered to be the safest approach for protecting users, whereas “limited” access to a shared facility of improved design is considered less safe. Shared latrines have historically been considered unimproved, based on the premise that accessibility, hygiene maintenance, and safety may be of low quality and may not elicit sufficient use to prevent environmental fecal contamination.^9^ Consistent with this policy, sharing a sanitation facility with just a few other households has been repeatedly associated with increased diarrhea risk in children and adults, compared with the use of private sanitation facilities.^10,11^ Based on this classification system, an estimated 638 million people using shared facilities of an otherwise improved design lacked access to an improved sanitation facility in 2015.^8^ Shifting these people from shared to household sanitation is unlikely to change quickly as lack of space and cost are key barriers to owning a private household latrine in poor urban areas.^12^

The classification of shared sanitation facilities as unimproved is controversial though. Policy on what constitutes improved sanitation has been limited by a lack of evidence about whether household latrine safety and use of latrines for safe disposal of feces differ based on the type of shared sanitation access.^9^ Latrines that are considered unsanitary, unsafe, and costly or that lack privacy are often avoided, resulting in open defecation. Public or communal latrines are more likely to be perceived as unsafe, unhygienic, and inaccessible, compared with minimally shared latrines, and public latrine users are notoriously inconsistent in latrine usage.^12–17^ Concerns about safety may decrease the use of public latrines by women, and social or economic limitations or inconvenience of access may reduce their use for child feces disposal.^18,19^ By contrast, households with consistent, inexpensive access to a latrine in the compound—regardless of being shared among neighbors or private—may be more consistent in using the latrine for all feces disposal practices to maintain household hygiene. Some even argue that latrines shared by a minimal number of households provide similar levels of accessibility, safety, and cleanliness as private latrines and can be a practical, inexpensive alternative for increasing sanitation coverage where private latrines are currently unfeasible.^9^ Although shifting households up the sanitation ladder from public to minimally shared latrines would likely improve privacy, dignity, and safety for users, it remains unclear whether this could improve safe disposal of excreta and reduce transmission of the enteric pathogens that contribute to the global pediatric diarrheal disease burden.

The objective of this study was to test the hypothesis that low-income urban households with access to a within-compound latrine are more likely to use latrines for child defecation and child feces disposal compared with households that rely on public latrines. Young children typically use diapers or child-sized potties, rather than latrines designed for adults, or are allowed to freely defecate in the open because of the perception that child feces are safe.^20,21^ Even in households where latrines are used by adults, disposal of child feces in the open is common, especially at night, when the latrine is far from the household or when the child is between 0 and 3 years of age.^10,22,23^ Thus, household child feces disposal practice is an useful indicator for identifying differences in overall household safe feces disposals based on the type of shared sanitation access. In addition, we examined whether perception of latrine use for child feces disposal is more common among neighboring households that share a compound latrine compared with neighboring households that use public latrines. Last, we tested whether we could detect differences in *Escherichia coli* and human adenovirus contamination on household surfaces in households with versus without a within-compound latrine.

## METHODS

### Study site.

This study is a part of the SaniPath study of fecal exposure in low-income urban Accra, Ghana. Accra was selected for this study because Ghana has one of the lowest rates of sanitation coverage worldwide, with approximately five million Ghanaians, equivalent to approximately 19% of the country’s population, practicing open defecation and another 16 million (approximately 66%) using unimproved or shared facilities.^8^ Data were collected in four low-income, non-adjoining neighborhoods in Accra: Alajo, Bukom, Old Fadama, and Shiabu.^12^

### Ethical considerations.

The study protocol was approved by the Institutional Review Board at Emory University, GA (protocol number: IRB00051584) and the University of Ghana Noguchi Memorial Institute for Medical Research Institutional Review Board (protocol number: IRB00001276). Participants were informed of the study objectives, and written informed consent for household data collection was obtained from adult household participants. Consent and interviewing were performed in English or the participant’s native language.

### Survey data collection and management.

Population-based surveys were conducted using paper-based forms in 844 households between March and November 2012. Sampling areas were identified by selecting four areas in each neighborhood using satellite maps. Within these areas, enumerators randomly selected a compound at the edge of each survey area by coin toss and identified one household with a child less than the age of 12 for enrollment in the study. A household was defined as persons who shared cooking and living arrangements, and a compound was defined as one or more households sharing a communal yard. After obtaining consent for participation in the study, trained data collectors administered a household survey to the primary caregiver or head of the household to record sociodemographic information and household water, sanitation, and hygiene access and practices. In most cases, the survey respondent was a woman. Sanitary inspections were conducted to verify the presence or absence of water and sanitation facilities, the condition of the facility, and visual observation of the household for feces on the ground. Subsequent households were selected by approaching every fifth compound on the street, counting both sides. In addition, 44 households with at least one child less than 12 years of age were recruited with assistance of a neighborhood liaison for participation in a structured observation study that included collection of hand rinses and surface swabs in the household for microbial testing. Data were entered using Microsoft Access and cleaned using SAS 9.4. Anonymity was maintained through the use of household identification numbers.

### Sanitation access and usage variables.

Preliminary descriptive analyses of household sanitation access revealed few households with a private basic facility (Supplemental Table 1), so households with private and compound-shared access were combined as “within-compound latrine” for comparison to households that reported relying on public latrines (Table 1).^8^ All households participating in this study were located in a neighborhood served by one or more public latrines.^12^ In households with a child between 5 and 12 years of age, the survey recorded whether the child defecated in any latrine versus an open location. In households with a child < 5 years of age, two variables for feces management of children < 5 years were created to represent where children defecated and where the feces were ultimately discarded. Preliminary analysis indicated that the use of latrines by children < 5 years was uncommon (< 5%), especially when stratified by neighborhood or other variables. Therefore, child defecation location for a household was classified based on whether children reportedly defecated in a latrine, potty, or diaper versus an open ground. Preliminary analysis of where feces were discarded indicated that leaving feces on the ground was rare (1.2%) and disposal in rubbish or open drains was extremely common. A feces disposal variable was created that classified households based on disposal of child feces in a latrine with a septage pit versus other locations. Participants living in a household with a shared yard (“compound”) were asked about their perceptions of whether other mother(s) in their compound used potties to capture child feces and whether other mothers in the compound leave/dispose child feces on the ground. Field staff also visually inspected the household and yard and recorded whether human feces were observed on the ground.

**Table 1:** Coding of reported sanitation access, use by child demographic groups, and hygiene conditions in households in four low-income urban neighborhoods in Accra, Ghana

Sanitation exposure	Levels of indicator
Sanitation access	Within-compound improved latrine*
	Public facility
Defecation location in the compound of children aged 5–12 years	Children in the compound defecate in a household, compound, or public latrine
	Children do not use facility, but defecate in open
Defecation location of children aged < 5 years	Children defecate in potties, diapers, or latrines
	Children defecate on ground or in drain
Caretakers’ disposal location for feces from children aged < 5 years	Feces disposed in latrine
	Feces left or thrown on ground or disposed of in open drains or rubbish
Perception that other mothers sharing the same compound use diapers, potties, or latrines to capture feces for children aged less than 5 years vs. permit open defecation	Yes
	No
Perception that other mothers in the same compound leave feces for children aged less than 5 years on ground in the compound vs. dispose elsewhere	Other mothers do not leave feces on ground in the compound
	Other mothers leave feces on ground in the compound
Human feces observed on ground in the compound	Yes
	No

* Within-compound access includes households with private (“safely managed” or “basic” according to the WHO/UNICEF Joint Monitoring Program [JMP]) or shared (“limited” according to the WHO/UNICEF JMP) improved latrines.

### Environmental sample collection and processing.

Field teams collected up to two swabs and hand rinses per household, depending on the availability of subjects and objects touched by children. Hand rinses were collected by asking both caretaker and child study participants from each household to submerge each hand up to the wrist in sterile 500 mL phosphate buffered saline (PBS) in a Whirl-Pak bag. Hand surfaces were gently massaged from the outside of the bag for 30 seconds, and then repeated for the second hand. Alcohol-sterilized framing squares and premoistened macrofoam swabs (EnviroMax Swabs, Puritan Medical, Guilford, ME) were used to swab a 100-cm^2^ area of a floor, wall or furniture, or an entire irregular-shaped object that children contacted during structured observation periods. The eluate from swab samples was obtained by vortexing swabs twice in 4 mLs of PBS, pH 7.2 with 0.04% Tween-80.

### Microbial analyses.

*Escherichia coli* bacteria were used as indicators of overall environmental fecal contamination. Human-specific adenovirus was used as an indicator of human fecal contamination.^24,25^ *Escherichia coli* were enumerated by membrane filtration of three serial dilutions of the swab eluate (1 mL, 0.1 mL, and 0.01 mL) and three serial dilutions of hand rinse eluate (100 mL, 10 mL, 1 mL) using Environmental Protection Agency method 1604.^26^ Concentration of colony-forming units (cfu) per object or set of hands was estimated based on colony count; concentrations for samples with no colonies were imputed by replacement with 0.5× the lower limit of detection (LLOD), and those with colonies too numerous to count were replaced by 1.5× the upper limit of detection. Adenovirus DNA was extracted from 1.5 mL of the swab eluate using the FastDNA SPIN Kit for soil (MP Biomedicals, Solon, OH) plus five freeze–thaw cycles to burst viral particles. Duplicate 5-μL volumes of DNA were first tested using the QuantiFast Pathogen + Internal Control PCR kit (Qiagen, Valencia, CA) to identify virus-positive samples and samples with poor amplification due to inhibition. Samples with positive amplification in one or both duplicate reaction tubes, or that showed signs of inhibition, were quantified by real-time qPCR using the OneStep RT-PCR Kit (Qiagen, Valencia, CA) and a standard curve generated from a quantified stock of human adenovirus.^27^ Concentrations for samples with no detected adenovirus at either stage of screening were replaced by 0.5× the LLOD of the assay (50 genomic copies [gc] for OneStep and 5 gc for Quantifast), and duplicates were screened for consistency (difference ≤ 5 cycle threshold [CT]) to ensure that within-sample variance did not skew the viral concentration estimates. Concentrations for each sample were estimated by averaging the concentration of duplicates and back calculating to estimate the concentration per total eluate from a swabbed surface. Censored concentration data were logarithmically transformed to obtain a log_10_-normal distribution.

### Statistical analysis.

Descriptive statistics of household sociodemographics and sanitation conditions were generated using SAS 9.4. Principal component analysis (PCA) of eight household assets was used to generate a household wealth index using the PROC FACTOR command. Bivariate logistic regression was performed for the household latrine access variable and each outcome indicator of latrine use. Each model was then adjusted for *a priori* confounder variables that included neighborhood, religion, tenancy, education of caretaker, wealth index, number of persons living in the household, and household water access. Final reported adjusted effects are the odds ratio (OR) and 95% confidence intervals (CIs) for observing a latrine usage indicator in households with a latrine in the compound versus public latrine access. Because of anticipated variability in neighborhood-level latrine coverage, an interaction term for household latrine access and neighborhood was included in each model to test whether neighborhood-level coverage modified the association between household latrine access and each latrine use indicator. Interaction terms were removed from final adjusted models unless significant at *P* < 0.05.

The Shapiro–Wilk test and visual assessment of the normal probability plot of the log_10_-transformed concentrations of *E. coli* and adenovirus in hand rinse and swab samples suggested that adenovirus concentrations were not normally distributed. For consistency, nonparametric Wilcoxon signed-rank tests were used to test the null hypothesis that the population mean ranks of log_10_ concentrations of *E. coli* and adenovirus in hand rinse and surface samples differed between households based on the type of latrine access or latrine usage indicator responses.

## RESULTS

### Household sociodemographic characteristics by neighborhood.

Complete survey data were available for a total of 785 households. Level of education of the household head, tenancy status, religion, wealth index, proportion of households living in a compound with other households, latrine access, primary drinking water source, and animal presence varied in households recruited from each neighborhood (Supplemental Table 1). A quarter of households reported household access to a private or shared improved latrine in the compound, although this varied across neighborhoods, with the greatest access in Alajo (52.7%, *N* = 205) and Shiabu (37.8%, *N* = 175) and the lowest in Bukom (6.9%, *N* = 204) and Old Fadama (1.5%, *N* = 201). Access to a private household latrine was rare (21 households, 2.7%). Three-quarters of households reported that they relied on pay-per-use public latrines located in their neighborhood. When pooling across neighborhoods, comparison of *a priori* selected potential confounders between households with within-compound (private and shared) latrines versus households that rely on public latrines indicated that higher levels of education and wealth, and Christian religion were more common in households with within-compound versus public latrine access (Table 2). Tenancy, number of persons living in the household, number of households living in the compound, drinking water source, and presence of animals in the household were not significantly different for households with within-compound versus households that reported using public latrines.

**Table 2:** Sociodemographic characteristics for 785 urban households in four low-income neighborhoods of Accra, Ghana, by reported compound and public latrine access level

Sociodemographic characteristics	Within-compound latrine,* *N* = 199	Public latrine, *N* = 586	*P* value†
Education of caregiver, % (*n*)			
No formal education	9.1 (18)	25.8 (151)	< 0.0001
Completed primary	18.1 (36)	27.8 (163)	
Completed secondary or higher	72.9 (145)	46.4 (272)	
Tenancy status (own), % (*n*)	62.3 (124)	62.5 (366)	0.97
Religion, % (*n*)			
Christian	82.4 (164)	70.5 (413)	0.004
Muslim	17.1 (34)	27.8 (163)	
Other	0.5 (1)	1.7 (10)	
Proportion of households sharing a compound with other households, % (*n*)	79.6 (163)	82.5 (442)	0.33
Number of people in a household, mean (SD)	5.2 (3.6)	6.4 (41.2)	0.67
Wealth index, mean (SD)‡	0.44 (0.64)	−0.11 (1.04)	< 0.0001
Water source, % (*n*)			
Sachet	78.4 (156)	78.0 (457)	0.96
Municipal piped water	21.1 (42)	21.3 (125)	
Stored piped water	0.5 (1)	0.7 (4)	
Animal presence in HH, % (*n*)	33.7 (67)	27.7 (162)	0.11

* Within-compound access includes households with private (“safely managed” or “basic” according to the WHO/UNICEF Joint Monitoring Program [JMP]) or shared (“limited” according to the WHO/UNICEF JMP) improved latrines.

† *P* value for differences in number (percentage) of households from χ^2^ distribution and from analysis of variance for mean and standard deviation (SD).

‡ Accounted for 28% of variance in wealth in this population.

### Relationship between latrine access and usage indicators.

After adjusting for each of the potential confounders, access to a within-compound latrine was associated with higher latrine use for defecation by children of 5–12 years of age compared with households relying on public latrines in 399 households with children in this age range (adjusted odds ratio [aOR] 2.62; 95% CI 1.00, 6.90; Table 3). Among 398 households with children < 5 years, access to a within-compound latrine was not associated with the use of diapers, potties, or latrines by children < 5 versus defecation on the ground (aOR 1.61; 95% CI 0.17, 15.08) but was significantly associated with greater use of a latrine (versus open ground) for disposal of child feces (aOR 2.78; 95% CI 1.53, 5.03).

**Table 3:** Unadjusted and adjusted odds of household feces disposal practices based on the type of latrine access in four low-income urban neighborhoods of Accra, Ghana

Latrine usage practices	Within-compound latrine, % (*n*/*N*)	Public latrine, % (*n*/*N*)	Unadjusted OR (95% CI)	Adjusted OR (95% CI)*
Use of latrines vs. open defecation for children between 5 and 12 years of age, *N* = 399†	93.8 (91/97)	82.5 (249/302)	**3.23 (1.34, 7.76)**	2.62 (1.00, 6.90)
Use of latrines, potties, or diapers vs. open defecation by children < 5 years of age, *N* = 398‡	98.7 (76/77)	95.6 (307/321)	3.47 (0.45, 26.8)	1.61 (0.17, 15.1)
Disposal of child feces in latrine vs. open drain or ground, trash, *N* = 398‡	52.0 (40/77)	34.0 (109/321)	**2.11 (1.28, 3.49)**	**2.78 (1.53, 5.03)**
Perception that other mothers in the same compound use potties for child defecation vs. open defecation, *N* = 468§	65.0 (102/157)	68.2 (212/311)	0.87 (0.58, 1.30)	0.71 (0.44, 1.15)
Perception that other mothers in the same compound leave child feces on ground vs. dispose elsewhere, *N* = 520§	5.3 (9/171)	10.9 (38/349)	**0.46 (0.22, 0.96)**	0.71 (0.30, 1.72)
Human feces observed on ground in the compound vs. not observed, *N* = 785	5.0 (10/199)	2.6 (15/586)	2.01 (0.89, 4.56)	2.99 (1.00, 8.94)

CI = confidence interval; Ref. = reference; OR = odds ratio. Bold reflects association significant at *P* < 0.05. Proportion of households reporting a latrine usage practice are reported as % and number out of total number of households in the on-site or public latrine group.

* Adjusted models include variables for household neighborhood, religion, wealth index, education of child caregiver, number of persons living in the household, and tenancy status.

† Children aged between 5 and 12 years in the household.

‡ Children aged < 5 years in the household.

§ Households that share a compound yard with other households.

Most households (79% of 785) shared a compound yard with other households for cooking, child play, and other domestic activities (Table 2). Therefore, communal spaces used for domestic purposes by these households could be contaminated by unsafe feces management practices of neighbors—regardless of the safety of their own behaviors. To test the hypothesis that within-compound (versus individual household) latrine access could improve feces disposal practices throughout a compound, study households were asked about their perceptions of defecation and feces disposal practices for within-compound neighbors with children aged < 5 years. The presence of a within-compound latrine was not associated with the perceived use of potties or diapers by other mothers in the compound for capturing child feces (aOR 0.71; 95% CI 0.44, 1.15; Table 3) versus mothers in the compound using public latrines. Respondents in compounds with within-compound latrines were less likely to report that other mothers in the same compound leave child feces on the ground in the compound compared with respondents in compounds relying on a public latrine. However, within-compound versus public latrine access was not associated with perceived neighbor feces disposal practices after adjusting for confounders (aOR 0.71; 95% CI 0.30, 1.72). Observation of human feces on the ground within the compound was rare, but was more common in compounds with within-compound latrines (5%) compared with compounds where all households used public latrines (2.6%) (aOR 2.99; 95% CI 1.00, 8.94). There was no evidence of effect modification by neighborhood on the association between household latrine access and latrine usage practices, so interaction terms were not included in final models.

### Differences in *E. coli* and human adenovirus fecal indicators on household surfaces and hands by sanitation access and use indicators.

**Figure 1.**
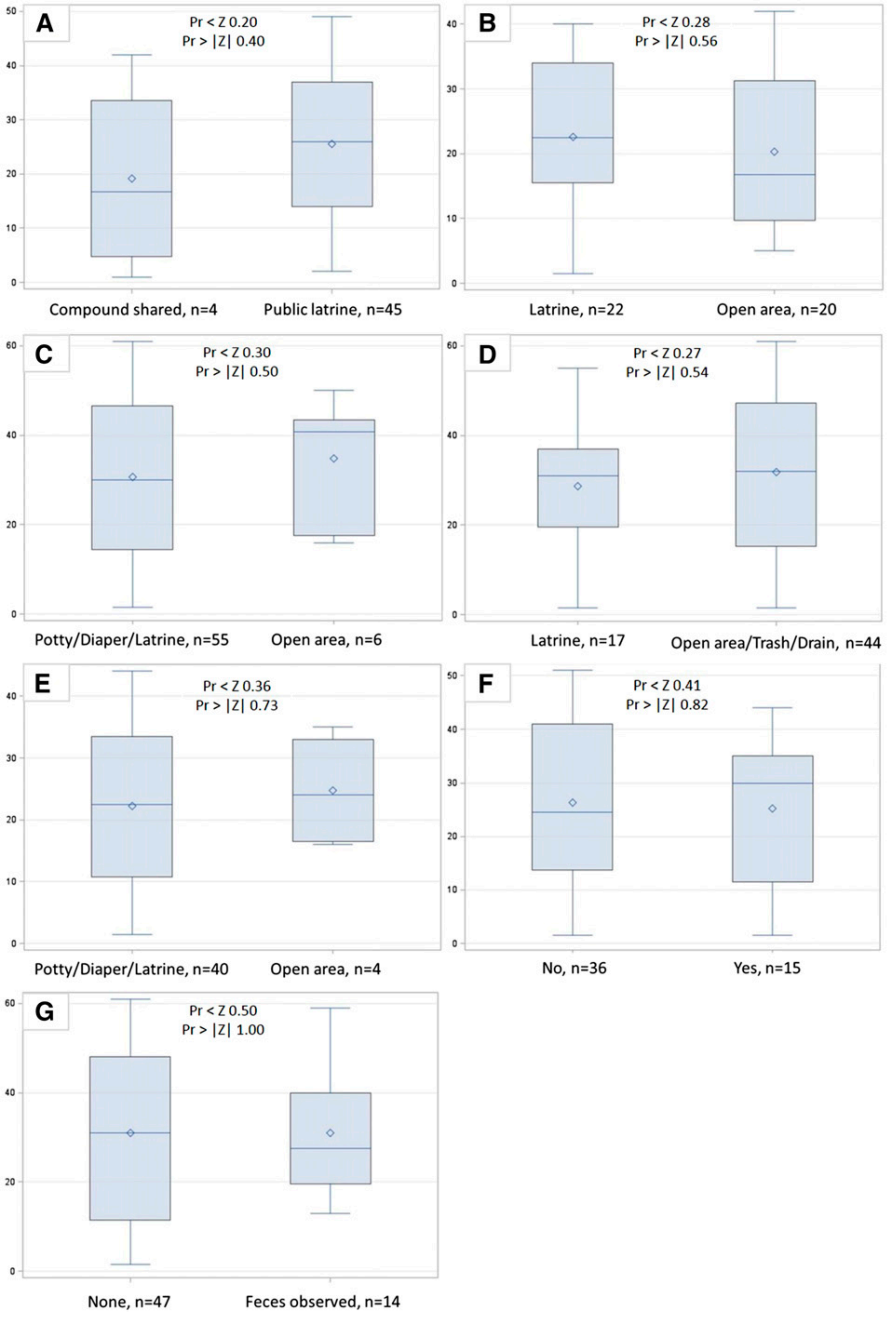
Wilcoxon rank sum score boxplots of log_10_ *Escherichia coli* colony-forming units (cfu) per set of hands by (A) level of shared latrine access, (B) defecation location by children 5-12 years, (C) defecation location by children <5 years, (D) disposal location for child feces by caregivers, (E) use of potties by neighbors in compound for <5 child defecation, (F) neighbors in compound leave child feces on ground, and (G) observed human and animal feces on ground. This figure appears in color at www.ajtmh.org.

**Figure 2.**
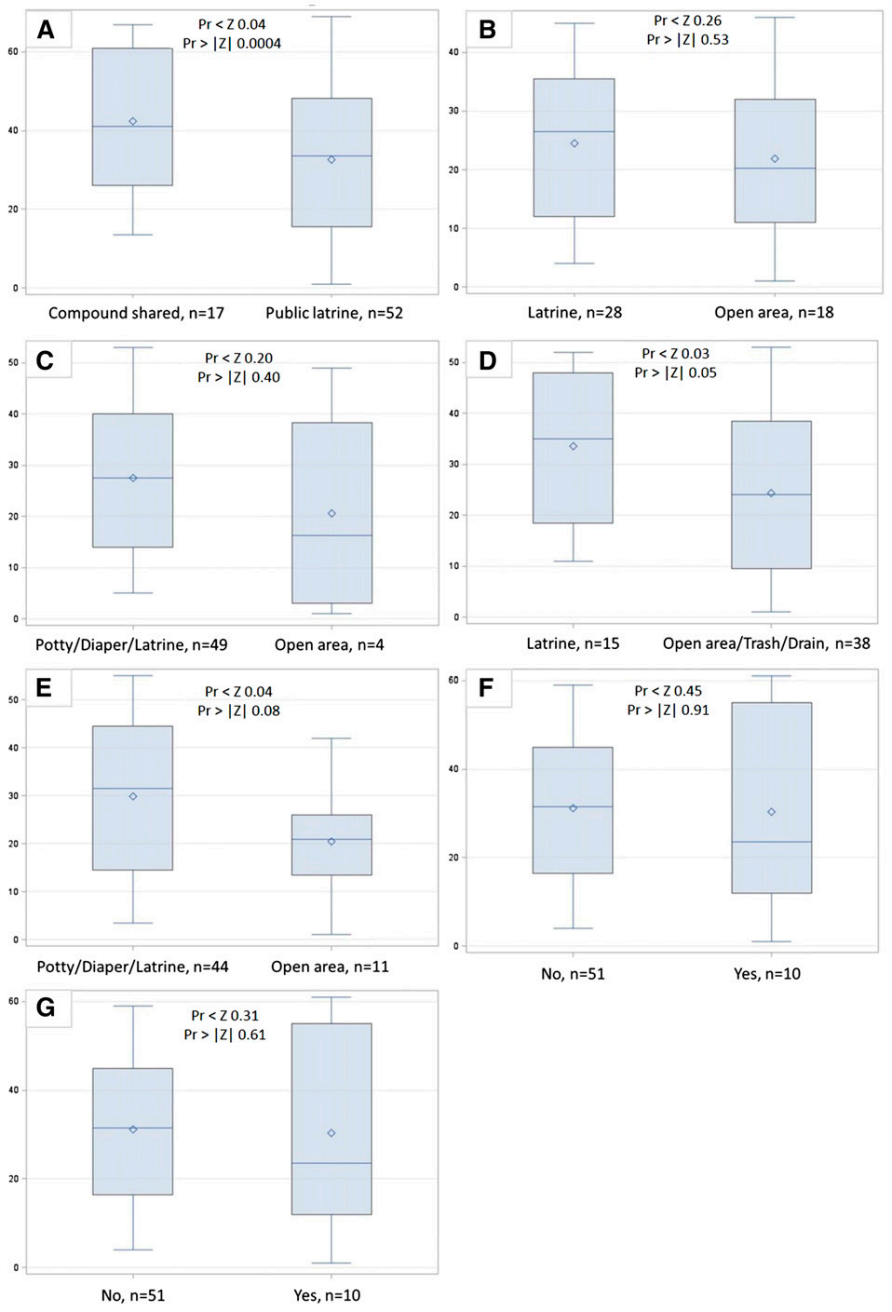
Wilcoxon rank sum score boxplots of log_10_ *Escherichia coli* colony-forming units (cfu) per 100 cm^2^ surface area by (A) level of shared latrine access, (B) defecation location by children 5-12 years, (C) defecation location by children <5 years, (D) disposal location for child feces by caregivers, (E) use of potties by neighbors in compound for <5 child defecation, (F) neighbors in compound leave child feces n ground, and (G) observed human and animal feces on ground. This figure appears in color at www.ajtmh.org.

**Figure 3.**
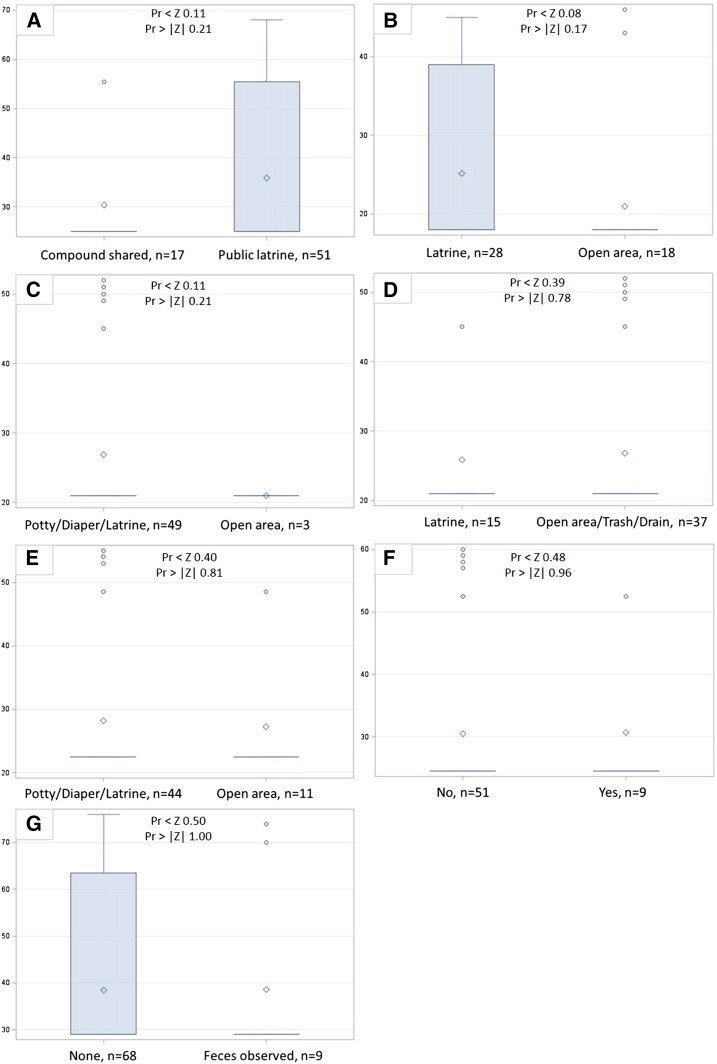
Wilcoxon rank sum score boxplots of log_10_ human adenovirus genomic copies (hAdv) per 100 cm^2^ surface area by (A) level of shared latrine access, (B) defecation location by children 5-12 years, (C) defecation location by children <5 years, (D) disposal location for child feces by caregivers, (E) use of potties by neighbors in compound for <5 child defecation, (F) neighbors in compound leave child feces on ground, and (G) observed human and animal feces on ground. This figure appears in color at www.ajtmh.org.

*Escherichia coli* were detected in 91.8% of 61 hand rinse samples with a mean of 2.5 log_10_ colony-forming units (cfu)/pair of hands (standard deviation [SD] = 1.14). *Escherichia coli* were detected in 88.3% of 77 household swab samples with a mean of 2.0 log_10_ cfu/cm^2^ (SD = 0.95). Human adenovirus was detected in 25% of 76 household swab samples with a mean of 2.4 log_10_ genomic copies (gc)/cm^2^ (SD = 1.2). *Escherichia coli* and adenovirus concentrations on household swab samples were poorly correlated (*r* = −0.1, *P* = 0.2). Wilcoxon rank sum scores of log_10_ *E. coli* concentrations on hands or household surfaces were not significantly different (two-sided *P* value < 0.05) for any comparison of latrine access or usage indicators (Figures 1–2). Log_10_ human adenovirus concentrations on household surfaces were also not significantly different between households with different latrine access or usage, although this may be due to low overall detection rates (Figure 3).

## DISCUSSION

To our knowledge, this is the first study to compare reported child feces disposal practices, based on household access to compound versus public shared latrines. In addition, the examination of perceived feces disposal practices for within-compound neighbors with compound or public latrine access was novel. Low rates of latrine use for child feces disposal were expected overall because of low global levels of latrine use for child feces in similar settings.^28^ However, we hypothesized that latrine usage for child feces disposal would be more common in households with within-compound versus public latrine access because of potential contextual differences in convenience of access, safety, cost, and privacy.^12^ After adjusting for potential socioeconomic confounders and neighborhood,^10,15^ we observed that self-reported use of latrines by older children (5–12 years) and disposal of the feces of young children (< 5 years) were more common in households with a within-compound latrine than in households relying on public latrines. Although reported latrine usage was greater in households with minimally shared compound latrines versus public latrine access, feces were observed on the ground more often within households with latrines in the compound versus households with public latrine access. Observation of human feces on the ground in compounds was uncommon, making it difficult to draw further conclusions about whether access to within-compound sanitation better prevents human fecal contamination of the household environment compared with public latrines.

The sanitary practices of neighbors that share a compound could impact hygienic conditions of communal living spaces where children eat and play, regardless of the safety of a household’s sanitary practices.^29^ If neighbors allow their children to defecate on the ground in the compound or do not safely dispose of child feces, shared spaces could become contaminated with feces. We hypothesized that the benefits of within-compound latrines would extend to improvement in latrine usage for child feces disposal by neighboring caregivers. In contrast, we hypothesized that latrine usage for child feces disposal would be less common among households in compounds where all rely on public latrines. Within-compound latrine access could strengthen social agreements between neighbors to collectively maintain hygienic conditions in shared living spaces.^29,30^ Our results suggest that women with compound latrine access were less likely to report that their neighbors in the compound left child feces on the ground, compared with reports from women in households where all households in the compound used public latrines. However, this relationship was not observed after adjusting for socioeconomic status and other sanitation factors, suggesting that perceived child feces disposal practices of within-compound neighbors are influenced more by education, wealth, or tenancy status than by the presence of a latrine. We could not find prior studies describing interactions between sharing of latrines and hygienic maintenance of shared living spaces. However, defined cooperative agreements among households sharing a latrine are an important determinant of latrine cleanliness.^31,32^ If social attitudes about cooperation in maintaining hygiene of all shared living spaces is similar to attitudes about maintaining latrines, then collective decision-making and having defined commitments among a limited number of households within a compound may also be a key determinant of preventing fecal contamination of the compound. For example, using latrines for disposal of child feces is expected and socially motivated among households in a compound. However, the benefits of within-compound latrine access in situations where *both* latrine and living spaces are intimately shared remain poorly understood.

Microbial indicator assays were used to examine and compare household fecal contamination for different types of latrine accesses and usage conditions. We found no significant differences in *E. coli* or human adenovirus concentrations on household surfaces when comparing households by within-compound versus public latrine access, or by latrine usage practices. However, the small sample sizes available for comparison and the low frequency of detection of human adenovirus in household environmental samples did not provide sufficient statistical power to compare within-compound versus publicly shared sanitation access. Furthermore, low levels of human adenovirus were insufficient to distinguish between human versus other sources of fecal contamination. One prior study used *E. coli* to distinguish between high versus low contamination areas in rural Tanzanian households, but overall *E. coli* levels were similar for households with improved and unimproved sanitation.^33^ If nonhuman fecal sources such as domestic animals (Table 2) or (unmeasured) adult defecation practices play a larger role in household *E. coli* contamination, then the possible impact from differences in child feces disposal practices may not be observed. Another possibility is that some portion of the *E. coli* detected on surfaces and hands may reflect naturalized populations of *E. coli* in the environment.^34–40^

This study design was cross-sectional and cannot establish causality. Because of the low frequencies of households with private within-compound latrines in these Accra neighborhoods, there were not a sufficient number of households to compare private latrines versus shared latrines. Although this is an analytical limitation for addressing policy questions about differences between “safely managed” or “basic” latrines versus “limited” shared latrines, latrine sharing is the norm in many urban settings, and the results from this study provide some insight into differences in types of shared sanitation within similar settings. Data on latrine access were visually confirmed, but actual use of latrines was based on self-reported practices. These practices may have been over- or underreported, based on the perception of acceptable behaviors in study neighborhoods. The household surveys were designed to capture the best available knowledge about factors that could confound the relationship between sanitation access and child feces management or fecal contamination of the household environment. However, the surveys may not have measured all the important determinants of child feces management, such as integrity, daily accessibility, and hygiene conditions of latrines or social relationships between households sharing a latrine.

These results suggest that child feces disposal practices are better in households in Accra with within-compound latrines compared with households that rely on public latrine access. Minimally shared compound-shared latrines may be a cost-effective alternative for increasing the number of households in Accra using a sanitation facility,^12^ which if safely managed, could reduce human-specific fecal contamination of the environment. However, these results also highlight that, in spite of the benefits of compound latrines for improved human waste containment, household fecal contamination was not completely eliminated. Thus, children in study households with compound-shared latrines could still be exposed to feces-transmitted pathogens. This may reflect insufficient levels of within-compound use of latrines by neighbors or insufficient levels of compound-level sanitation coverage within these neighborhoods to provide “herd-protection” from exposure to the feces of others.^41^ Alternatively, this may reflect limited benefits of latrine access where domestic animals also contribute to household fecal contamination. The data from this study could not be used to explore the relationship between sanitation access and observed feces in household yards because this was a rare occurrence. Yet, these results suggest that investment in minimally shared latrines will not eliminate fecal contamination of the household environment and consequently feces-transmitted diseases. One key research gap is how sharing of communal living spaces affects environmental hygiene and child health. Better microbial indicators are also needed for assessing the contribution of different fecal sources on environmental exposure risks for children.

## Supplementary Material

Supplemental Table
