# Supplementary material for: Within-Compound Versus Public Latrine Access and Child Feces Disposal Practices in Low-Income Neighborhoods of Accra, Ghana
Source: Am J Trop Med Hyg. 2018 Mar 19;98(5):1250–9. doi: 10.4269/ajtmh.17-0654 (PMC5953368; doi:10.4269/ajtmh.17-0654)
Supplement: Supplementary file 1 [file tpmd170654.SD1.pdf]

Supplemental Table 1

Sociodemographic characteristics for 785 urban households in four low-income neighborhoods of Accra, Ghana, by neighborhood

Neighborhood	Alajo *N* = 205	Bukom *N* = 175	Old Fadama *N* = 204	Shiabu *N* = 201	*P* value*
Education of caregiver, % (*n*)					
No formal education	14.2 (29)	15.4 (27)	47.6 (97)	8.0 (16)	< 0.0001
Completed primary	20.0 (41)	41.7 (73)	21.1 (43)	20.9 (42)	
Completed secondary or higher	65.9 (135)	42.9 (75)	31.4 (64)	71.1 (143)	
Tenancy status (own), % (*n*)	51.7 (106)	82.3 (144)	66.2 (135)	52.2 (105)	< 0.0001
Religion, % (*n*)					
Christian	77.6 (159)	89.1 (156)	33.8 (69)	96.0 (193)	< 0.0001
Muslim	22.4 (46)	8.0 (14)	64.2 (131)	3.0 (6)	
Other	0 (0)	2.9 (5)	2.0 (4)	1.0 (2)	
Households living in compound, % (*n*)	79.6 (163)	82.5 (144)	59.5 (121)	94.6 (190)	< 0.0001
Number of people in household, mean (SD)	5.26 (3.58)	5.67 (4.86)	8.97 (69.72)	4.53 (1.98)	0.61
Wealth index, mean (SD)†	0.28 (0.85)	−0.14 (1.15)	−0.34 (0.96)	0.31 (0.83)	< 0.0001
Facility access, % (*n*)*					
Private basic facility	3.2 (16)	0.01 (1)	0 (0)	2.0 (4)	< 0.0001
Compound-shared basic facility	44.9 (92)	6.3 (11)	1.5 (3)	35.8 (72)	
Public facility	47.3 (97)	93.1 (163)	98.5 (201)	62.2 (125)	
Water source, % (*n*)					
Sachet	74.6 (153)	71.4 (125)	92.2 (188)	73.1 (147)	< 0.0001
Municipal piped water	24.9 (51)	28.0 (49)	7.4 (15)	25.9 (52)	
Tap of polytank with stored piped water	0.5 (1)	0.6 (1)	0.5 (1)	1.0 (2)	
Animal presence in HH, % (*n*)	33.8 (69)	23.4 (41)	20.7 (42)	38.3 (77)	0.0002

SD = standard deviation.

* *P* value for differences across neighborhoods in number (percentage) of households from χ^2 distribution, and from ANOVA for mean and standard deviation.

† Wealth index calculated by principal component analysis of eight household assets using the PROC FACTOR command.
